# Evaluation of factors influencing expression and extraction of recombinant bacteriophage endolysins in *Escherichia coli*

**DOI:** 10.1186/s12934-022-01766-9

**Published:** 2022-03-15

**Authors:** Cecilia Lucía Balaban, Cristian Alejandro Suárez, Carina Andrea Boncompain, Natalia Peressutti-Bacci, Eduardo Augusto Ceccarelli, Héctor Ricardo Morbidoni

**Affiliations:** 1 Laboratorio de Microbiología Molecular, Facultad de Ciencias Médicas, Universidad Nacional de Rosario, Rosario, Argentina; 2 Instituto de Biología Molecular y Celular de Rosario, Consejo Nacional de Investigaciones Científicas y Técnicas, Rosario, Argentina

**Keywords:** Endolysins, Bacteriophages, *Staphylococcus aureus*, Chaperones, Recombinant expression, Solubility

## Abstract

**Background:**

Endolysins are peptidoglycan hydrolases with promising use as environment-friendly antibacterials mainly when used topically. However, in general, endolysin expression is hampered by its low solubility. Thus, a critical point in endolysin industrial production is optimizing their expression, including improvement of solubility and recovery from cell extracts.

**Results:**

We report the expression of two endolysins encoded in the genome of phages infecting *Staphylococcus aureus*. Expression was optimized through changes in the concentration of the inducer and growth temperature during the expression. Usually, only 30–40% of the total endolysin was recovered in the soluble fraction. Co-expression of molecular chaperones (DnaK, GroEL) or N-term fusion tags endowed with increased solubility (DsbC, Trx, Sumo) failed to improve that yield substantially. Inclusion of osmolytes (NaCl, CaCl_2_, mannitol, glycine betaine, glycerol and trehalose) or tensioactives (Triton X-100, Tween 20, Nonidet P-40, CHAPS, *N*-lauroylsarcosine) in the cell disruption system (in the absence of any molecular chaperone) gave meager improvements excepted by *N*-lauroylsarcosine which increased recovery to 54% of the total endolysin content.

**Conclusion:**

This is the first attempt to systematically analyze methods for increasing yields of recombinant endolysins. We herein show that neither solubility tags nor molecular chaperones co-expression are effective to that end, while induction temperature, (His)_6_-tag location and lysis buffer additives (e.g*.* *N*-lauroylsarcosine), are sensible strategies to obtain higher levels of soluble *S. aureus* endolysins.

**Supplementary Information:**

The online version contains supplementary material available at 10.1186/s12934-022-01766-9.

## Background

The rise in antibiotic resistant bacteria is a threat to human health due to the limited therapeutic options developed over the last years. The efficacy of the few new molecules that have been added to the antibacterial portfolio were, in most cases, quickly compromised due to bacterial resistance that arose and spread quickly. This situation calls for developing novel and radically different methods to counterattack bacterial infections [1, 2]. In this scenario, being outsmarted by microorganisms, the idea of using phages and their lytic enzymes as new defensive antibacterial materials has resurfaced. After a century of being reported for the first time, phages are still at the center of a debate about their utilization. This debate slows down the badly needed use of phage cocktails to treat human and animal infections caused by drug-resistant bacteria. Many opinions favor or oppose the use of phages. However, phage cocktails are already applied for non-human use [3, 4]. A possible way to overcome the stalemate on phage utilization is by supporting the use of endolysins, potent phage-encoded enzymes with peptidoglycan hydrolase activity. Although the chemical structure of peptidoglycan is relatively conserved, subtle changes in their composition allow for the grouping of bacteria according to their chemotypes, reflecting PG slight but important diversity. This explains why endolysins are able to discriminate and destroy the cell envelope of distinct bacterial species. A number of attributes of these peptidoglycan-hydrolases make them attractive candidates for classic antibiotic replacement, among them: narrow lytic spectrum, nanomolar binding affinity, relatively easy manipulation for custom design and unlikely evolution of resistance (since they target essential structures of cell wall). Endolysins are made at the late stage of phage infection and are expressed along with holins, proteins that, by making pores in the cell membrane, allow the endolysins to reach their peptidoglycan (PG) target [5, 6]. Of note, it has been demonstrated that endolysins from phages infecting Gram (+) bacteria are highly effective when added externally [7].

Over the past two decades, these enzymes were proven to have strong antibacterial activity against Gram-positive pathogens when applied extracellularly, and some of them are currently in clinical trials (CF-301, ClinicalTrials.gov, NCT04160468) or already in the biopharmaceutical market (Staphefekt™, https://www.staphefekt.com) [8]. Nevertheless, one of the main hurdles in recombinant production of endolysins is their low recovery in the soluble form. There are a number of reviews addressing endolysin features as alternative antimicrobials [9–11]; however, there is little or incomplete information on the reasons for their relatively low recovery from cell extracts at the bench scale. Despite that, at least one commercial use of an endolysin of Gram (+) phage origin has been reported [8], demonstrating that it is feasible to overcome the purification hurdle.

These endolysins have a modular structure consisting of a domain that determines the specific binding to the bacterial cell wall (CBD) and one or two enzymatically active domains (EAD) that can cleave the different bonds present in peptidoglycan. This modular structure gives an experimental frame to propose the design of tailor-made endolysins accommodating EADs with different specificities against one or more Gram (+) bacteria. Simultaneously, it creates a unique opportunity to develop enzymatic antibacterial molecules with improved killing activity [12].

However, a major unsolved problem is the low solubility of most of the reported endolysins [13–15], a point that could dissuade the industrial production of these enzymes. So far, there has not been any systematic approach to address the expression of endolysins and their recovery from the bacterial cell extract. A recent report by Zydsiecki et al*.* described the successful use of matrix assisted refolding of truncated forms of the endolysin LysK [16], a promising alternative worth addressing. Notwithstanding that, the adoption of such approach would require a comprehensive testing on full length endolysins.

A different approach is reported herein, addressing the effect on endolysin recovery of different growth and expression conditions, the co-expression of several molecular chaperones as well as the use of solubility tags fused to the endolysin encoding genes. Finally, the effect of the composition of the buffer for cell disruption, including osmolytes, detergents, and salts, was also analyzed.

## Results

### Sequence alignment and domain organization of endolysins

**Fig. 1:**
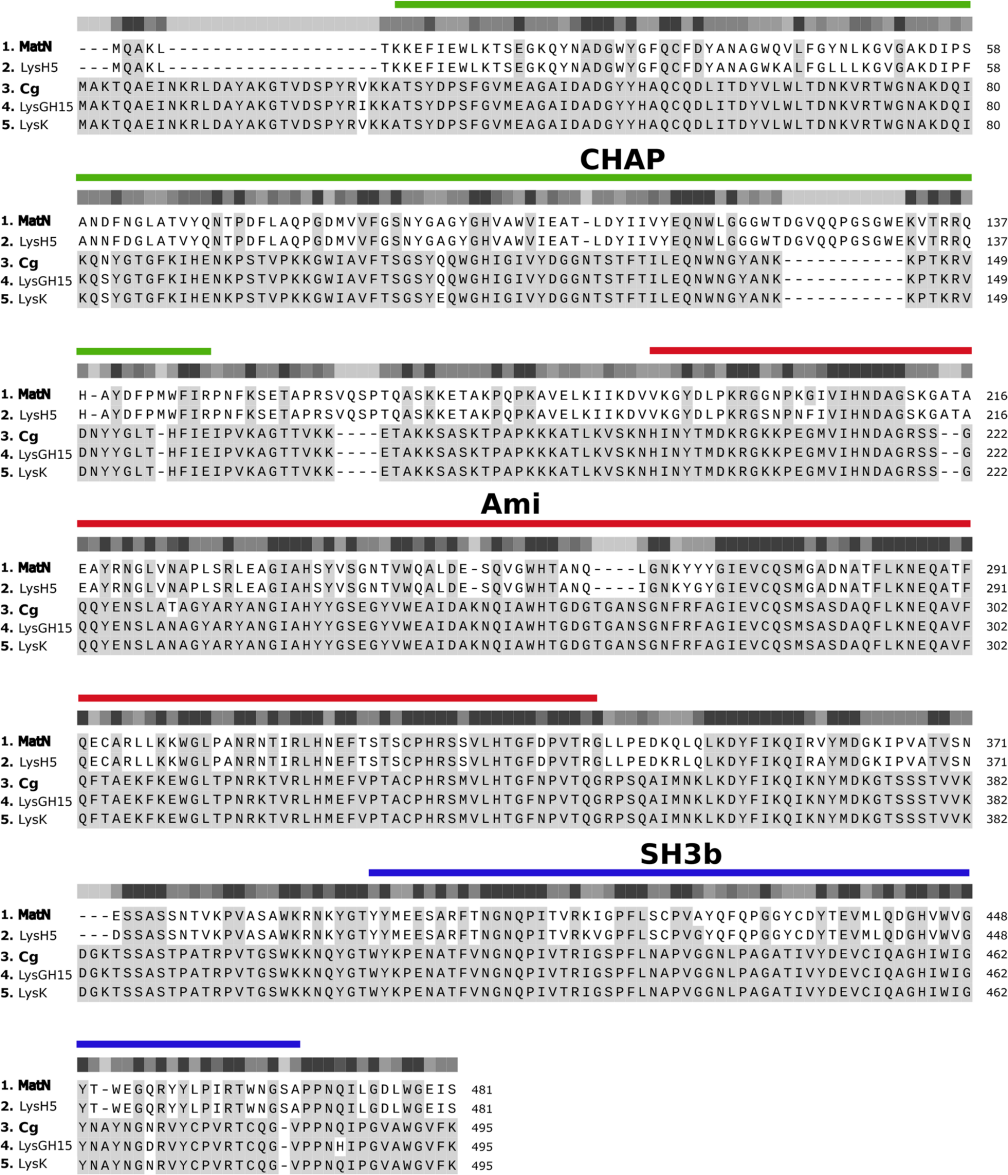
MatN and Cg domain architecture and alignment with reference endolysins. Prediction of the domain architecture was analyzed using InterPro. All endolysins are composed of a CHAP (cysteine, histidine-dependent amidohydrolase/peptidase; green), Ami (Amidase; red) and SH3b (blue) domain. Multiple alignment analysis of our endolysins MatN and Cg with endolysins previously reported in the bibliography (LysK, LysGH15 and LysH5) was done using Clustal Omega. Identical residues are shaded in gray and increasing degree of residue conservation correlates with darkening of the gray scale of the bar above the sequences

Cg and MatN were identified during the sequencing of bacteriophages active on *Staphylococcus aureus* isolated in our lab and chosen on the basis of their phylogenetic divergence (Suárez et al., submitted to Microbiology Spectrum, Spectrum00334-22). The amino acid sequences of Cg and MatN do not present remarkable differences when compared to the well characterized endolysins LysK, LysGH15 and LysH5 [14, 17, 18], showing high amino acid identity and similarity through their EADs (CHAP [IPR007921] and Amidase [IPR002502]) and CBD (SH3b [IPR003646]) as well as comparable domain organization (Fig. 1). Furthermore, hydrophobicity plots of these and other endolysins obtained with the Kyte-Doolittle scale showed no predominance of hydrophobic residues in any region of the amino acid sequence (Additional file 1).

### Effect of inducer concentration and induction temperature on the yield of soluble endolysin

**Fig. 2:**
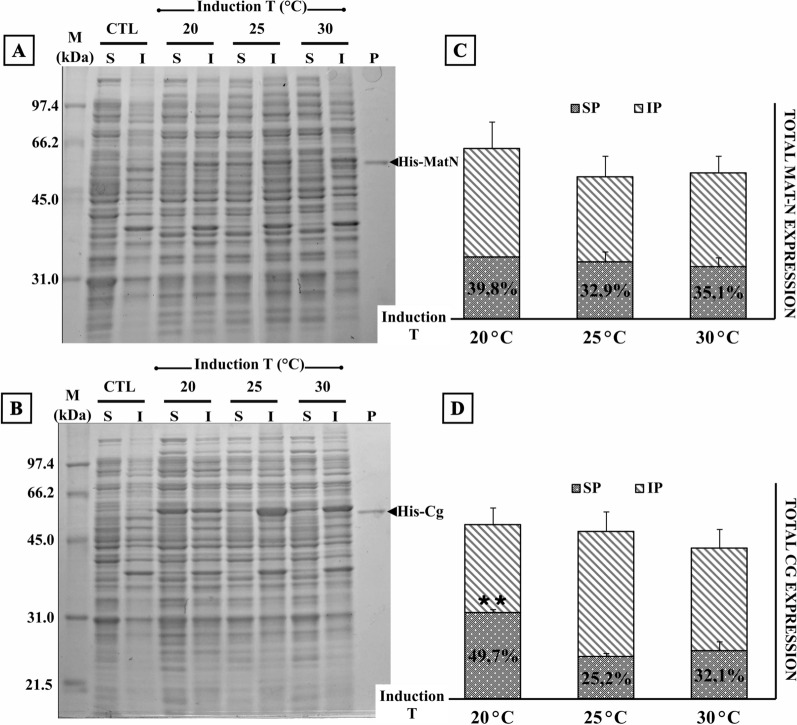
Effect of Temperature on recombinant endolysins MatN and Cg solubility and yield. **A, B** *E. coli* C43(DE3) harboring pET-32a-MatN/Cg was induced at 20, 25 °C, and 30 °C by addition of 1 mM IPTG. Cells were harvested after 20 h of incubation with constant shaking and processed to obtain soluble (S) and insoluble (I) fractions. Equivalent protein amounts (10 µg) of each sample were loaded on 12% SDS-PAGE. Upon gel electrophoresis, gels were stained with Coomassie Brilliant Blue R-250 and unstained following standard protocols. Control (CTL) lanes correspond to cellular lysates of uninduced cultures. A sample of purified (His)_6_-MatN/Cg endolysin (57 kDa/57.7 kDa) was loaded on P lane. **C, D** Total amounts of recombinant endolysin (column height) and the ratio of soluble/insoluble protein (SP, gray dotted pattern; IP, diagonal lines pattern) were calculated after background normalization with ImageJ, open-source image processing software. Endolysin expression and solubility were analyzed in biological triplicates. Error bars represent SD of mean values. **p  <  0.005 (soluble endolysin ratio of 20 °C group vs. higher induction temperature groups)

Since the vast majority of the reports on identification of novel endolysins neither provide complete information on the expression yields (Table 1) nor address the reasons behind the low solubility of endolysins, we studied the influence of different factors during the process of induction and lysis. When the effect of the growth temperature during the induction stage was analyzed, we found a clear and reproducible difference in endolysin production on induced cultures kept at low temperature (20 °C) compared to 25 °C and 30 °C (Fig. 2). Cg endolysin induction at 20 °C promoted a significantly higher proportion of soluble endolysin (*p* = 0.002) when compared to higher induction temperatures.

**Table 1:** Expression and recovery conditions of previously reported endolysins at bench scale

Endolysin	Description	Expression vector/*E. coli* strain	Induction conditions	Lysis buffer	Purification yields (mg/L of bacterial culture)
LysH5 [18]	Endolysin from phage ΦH5: N-terminal CHAP, a central amidase domain (*N*-acetylmuramyl-l-alanine amidase), and a C-terminal SH3b CBD	pRSETB (N-ter His-tag)/BL21(DE3)/pLys	1 mM IPTG/18 h—19 °C	20 mM NaH_2_PO_4_,500 mM NaCl, 20 mM Imidazole, pH 7.4	2 mg/L
Lys-phiSA012 [27]	endolysin from phage phiSA012: N-terminal CHAP, a central amidase domain (*N*-acetylmuramyl-l-alanine amidase), and a C-terminal SH3b CBD	pGEX-6P-2 (C-ter GST-tag)/BL21(DE3)	0.1 mM IPTG/O.N.—25 °C	50 mM Tris–HCl, 1 M MgCl_2_ and 10% NP-40	Not stated
plyGRCS [28]	endolysin from phage GRCS: N-terminal both CHAP and endopeptidase activity and a C-terminal SH3b CBD	pBAD24 (C-His-tag)/BL21 (DE3)	0,25% ara/O.N.—18 °C	Not stated	Not stated
LysGH15 [17]	Endolysin from phage GH15: N-terminal CHAP, a central amidase domain (*N*-acetylmuramyl-l-alanine amidase), and a C-terminal SH3b CBD	pET15b (N-ter His-tag)/BL21 (DE3) (Codon Plus)	1 mM IPTG/6 h—25 °C	20 mM PBS	Not stated
2638A [29]	Endolysin from phage 2638A: N-terminal M23 peptidase domain, a mid-protein amidase 2 domain, and a C-terminal SH3b_5 (SH3b) CBD	pET21a (C-ter His-tag)/BL21(DE3)	1 mM IPTG/18 h—10 °C	Not stated	Not stated
ClyS [15]	Quimeric endolysin: N-terminal catalytic domain of Twort phage endolysin and C-terminal CBD from phi13 phage endolysin	pJML6/DH5α	2% lactose/O.N.—30 °C	20 mM NaH_2_PO_4_, 1 mM DTT	Not stated
CHAP-AMIDASE [30]	Quimeric endolysin: N-terminal CHAP domain and C-terminal amidase-2 domain from Phage K endolysin (LysK). GS-His tag linker	pET-22b (pelB leader)/BL21 (DE3)	1 mM IPTG/4 h—37 °C	Not stated	8–12 mg/L
LysP108 [31]	Endolysin from phage P108: N-terminal amidase and C-terminal SH3b CBD	pET21a (C-ter His-tag)/BL21 (DE3)	0,1 mM IPTG/10 h—23 °C	Not stated	Not stated
LysDW2 [32]	Endolysin from phage DW2: N-terminal CHAP, a central amidase domain (*N*-acetylmuramyl-l-alanine amidase), and a C-terminal SH3b CBD	pQE-60 (C-ter His-tag)/XL1Blue	1 mM IPTG/5 h—37 °C	BugBuster^®^ protein extraction reagent, Millipore, Merk	Not stated (only CHAP truncated domain was soluble)
SAL-1 [33]	endolysin from phage SAP-1: SAL-1 differs from LysK at three residues (ILE- > VAL 26, GLN- > GLU 114, and HIS- > GLN 486)	pBAD-TOPO (no His-tag)/BL21 (DE3)	0.2 mM ara/10 h—19 °C	50 mM Na_2_HPO_4_ (pH 7.5), 10 mM EDTA, 1 mM DTT	80 mg/L
LysCSA13 [26]	endolysin from phage CSA13: N-terminal CHAP and C-terminal SH3-5 CBD	pET28a (N-ter His-tag)/BL21 (DE3)	0,5 mM IPTG/20 h–18 °C	50 mM NaH_2_PO_4_, 300 mM NaCl, pH 8.0	10 mg/L

*Ara* arabinose

**Fig. 3:**
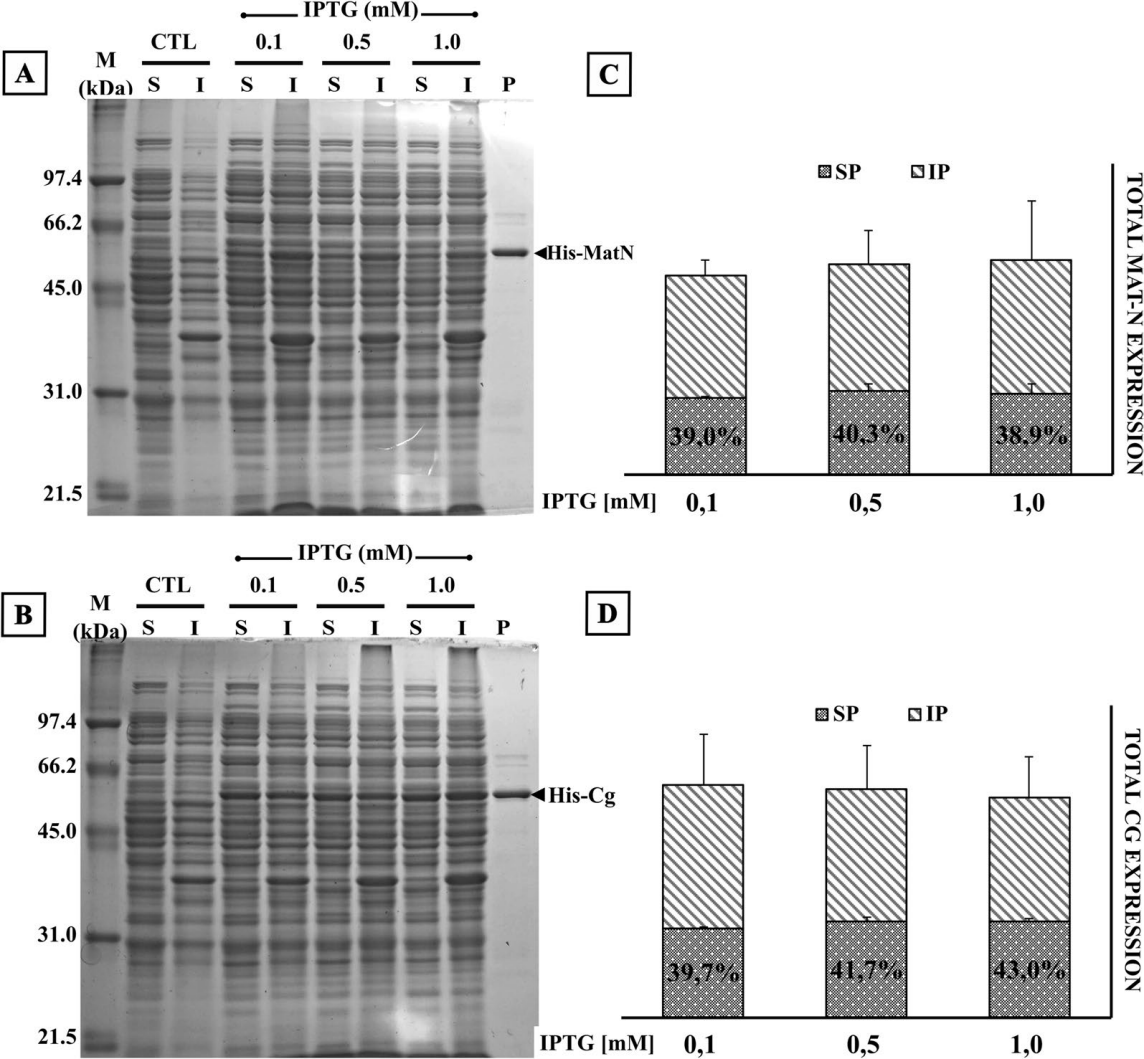
Effect of inducer concentration on recombinant endolysins MatN and Cg solubility and yield. **A, B** *E. coli* C43(DE3) harboring pET-32a-MatN/Cg was induced at 20 °C by the addition of three different IPTG concentrations (0.1; 0.5 and 1 mM). Cells were harvested after 20 h of incubation with constant shaking and processed to obtain soluble (S) and insoluble (I) fractions. Equivalent protein amounts (10 µg) of each sample were loaded on 12% SDS-PAGE. Upon gel electrophoresis, gels were stained with Coomassie Brilliant Blue R-250 and unstained following standard protocols. Control (CTL) lanes correspond to cellular lysates of uninduced cultures. A sample of purified (His)_6_-MatN/Cg endolysin (57 kDa/57.7 kDa) was loaded on P lane. **C, D** Total amounts of recombinant endolysin (column height) and the ratio of soluble/insoluble protein (SP, gray dotted pattern; IP, diagonal lines pattern) were calculated after background normalization with ImageJ, open-source image processing software. Endolysin expression and solubility were analyzed in biological triplicates. Error bars represent SD of mean values

Expression of the endolysin genes cloned in a pET-32a vector was induced by the addition of IPTG at various concentrations. Our results showed that the amount of endolysin produced did not increase at IPTG concentrations ranging from 0.1 to 1.0 mM. Moreover, we did not detect substantial changes in the amount of either soluble or insoluble endolysin (Fig. 3).

### The use of solubility tags does not improve endolysin solubility

**Fig. 4:**
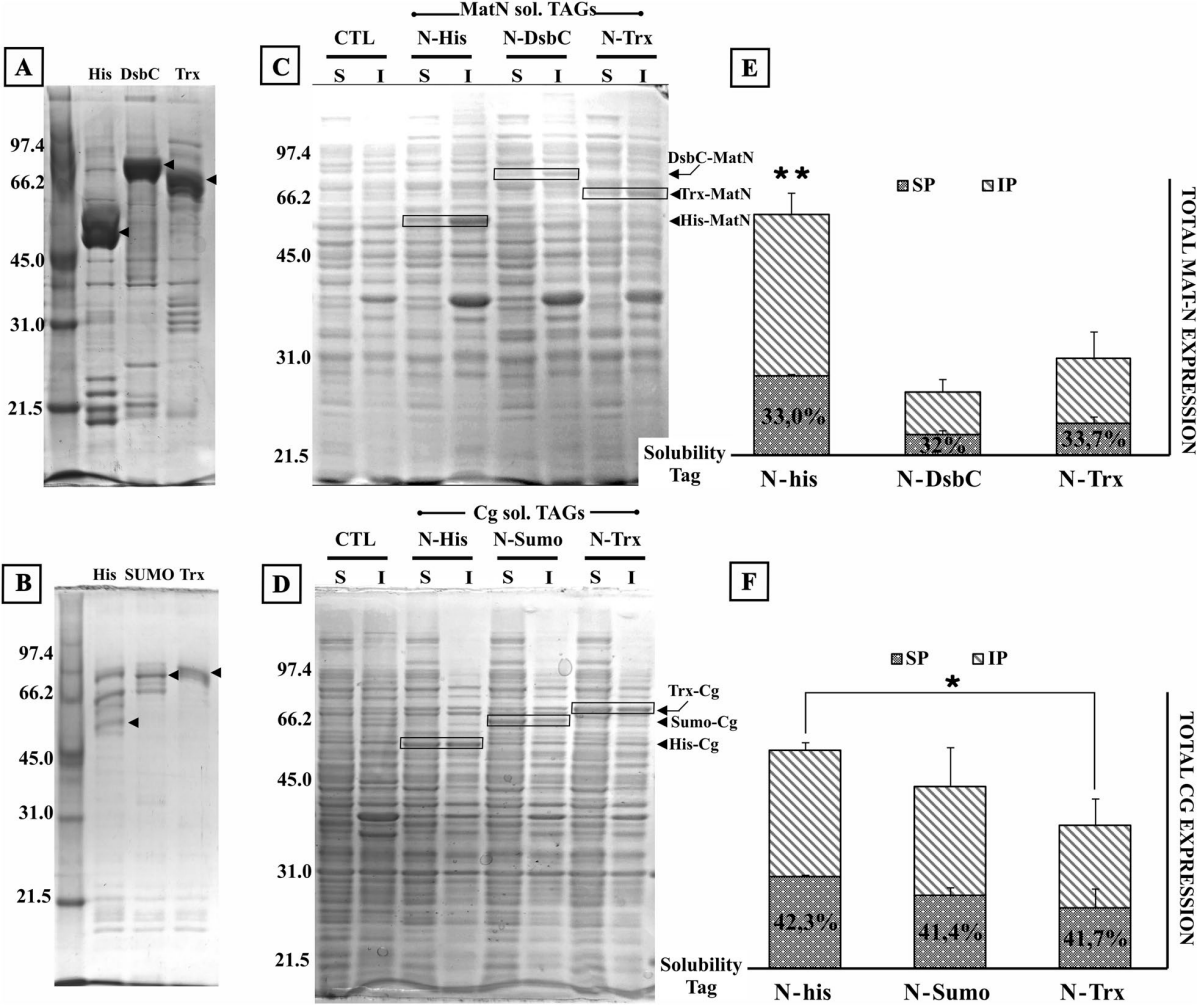
Solubility N-term tags performance on soluble endolysin yields. **A, B** SDS PAGE showing the position of each fusion tag to recombinant endolysins; **C, D** *E. coli* C43(DE3) harboring pET-32a modified with N-term solubility tags (DsbC, Sumo and Trx) in frame fusions to MatN/Cg genes was induced at 20 °C by addition of 1 mM IPTG. Cells were harvested after 20 h of incubation with constant shaking and processed to obtain soluble (S) and insoluble (I) fractions. Equivalent protein amounts (10 µg) of each sample were loaded on 12% SDS-PAGE. Upon gel electrophoresis, gels were stained with Coomassie Brilliant Blue R-250 and unstained following standard protocols. Control (CTL) lanes correspond to cellular lysates of uninduced *E. coli* C43(DE3) harboring pET32a-MatN/Cg. **E, F** Total amounts of recombinant endolysin (column height) and the ratio of soluble/insoluble protein (SP, gray dotted pattern; IP, diagonal lines pattern) were calculated after background normalization with ImageJ, open-source image processing software. Endolysin expression and solubility were analyzed in biological triplicates. Error bars represent SD of mean values. **p  <  0.005, *p  <  0.05 (total endolysin expression of N-His group vs. N-Tags groups)

To enhance the yield of soluble endolysin; we resorted to using three widely used solubility tags (DsbC, Trx and Sumo) [19–21] placed as N-term fusions to the endolysin genes. Surprisingly, despite DsbC being identified by high throughput screening as an efficient partner for recombinant protein solubilization, it did not increase endolysin content in the soluble fraction. On the contrary, this tag caused a reduction in total recombinant endolysin produced. Trx also caused a reduction in the synthesis of the recombinant enzyme although less drastically than DsbC. In this regard, (His)_6_-MatN total expression was on average 3 times higher than (His)_6_-DsbC/(His)_6_-Trx MatN, (*p* < 0.001), and (His)_6_-Cg total expression was 0.5 times higher than (His)_6_-Trx-Cg (*p* = 0.035). Thus, these two fusion constructs bearing N-term tags failed to produce a clear benefit in the level of soluble endolysin present in the cell extract (Fig. 4).

### (His)_6_ Tag localization affects MatN solubility

**Fig. 5:**
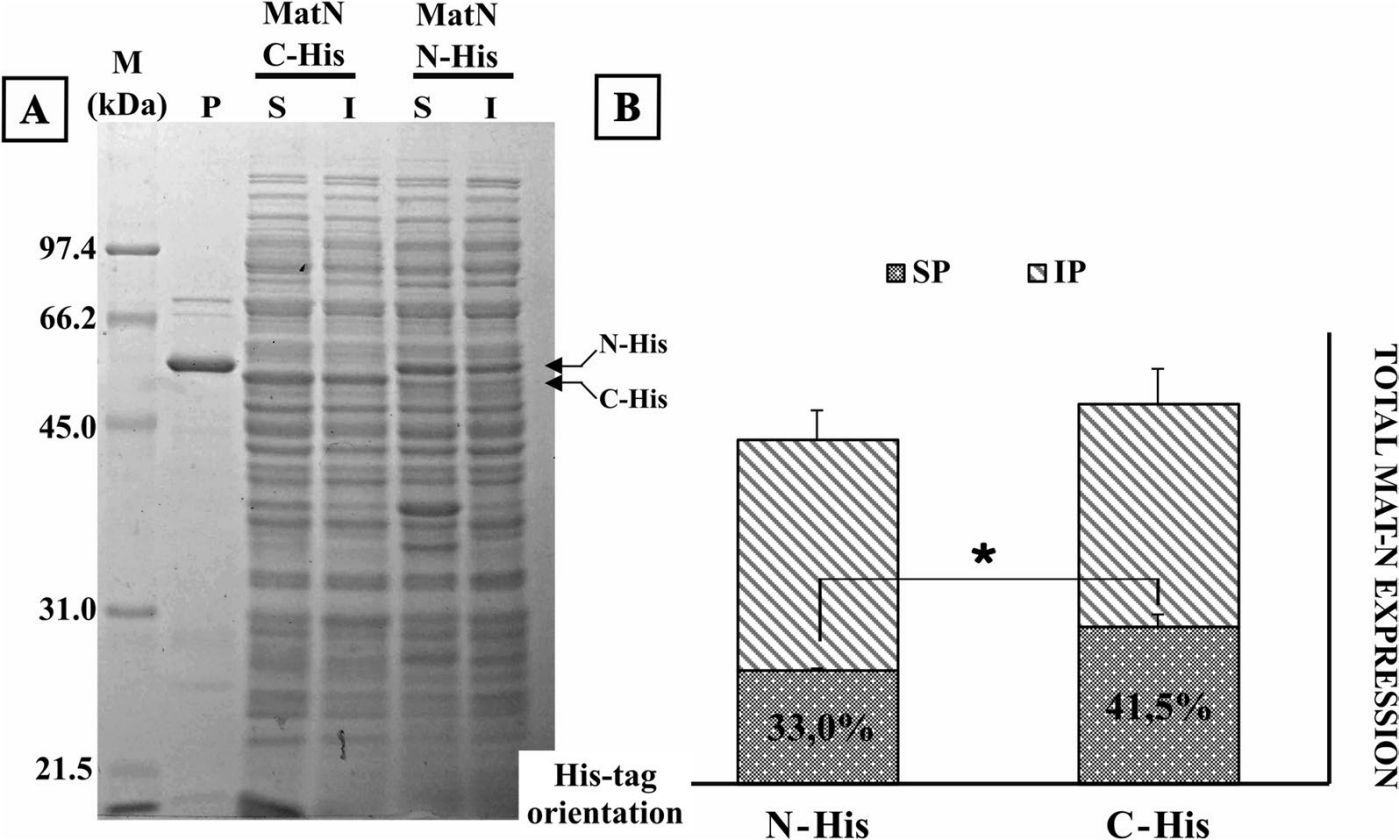
(His)_6_ tag orientation on MatN soluble yields. **A** *E. coli* C43(DE3) harboring pET-32a N-term- (his)_6_ MatN or pET-22b C-term-(his)_6_ MatN were induced at 20 °C by addition of 1 mM IPTG. Cells were harvested after 20 h of incubation with constant shaking and processed to obtain soluble (S) and insoluble (I) fractions. Equivalent protein amounts (10 µg) of each sample were loaded on 12% SDS-PAGE. Upon gel electrophoresis, gels were stained with Coomassie Brilliant Blue R-250 and unstained following standard protocols. A sample of purified N-term-His MatN endolysin (57 kDa) was loaded on P lane. **B** Total amounts of recombinant endolysin (column height) and the ratio of soluble/insoluble protein (SP, gray dotted pattern; IP, diagonal lines pattern) were calculated after background normalization with ImageJ, open-source image processing software. Endolysin expression and solubility were analyzed in biological triplicates. Error bars represent SD of mean values. *p  <  0.05 (comparison between soluble endolysin ratios)

Since the level of expression of a recombinant protein can vary when having either an N-term or a C-term (His)_6_ tag [22], we set to determine the effect of the former modification on the expression of MatN (Fig. 5). C-term (His)_6_ tag construct prompted a statistically significant increase of soluble endolysin when compared to the N-term bearing construction (*p* = 0.008).

### Co-expression of chaperone proteins does not improve yields of soluble endolysin

**Fig. 6:**
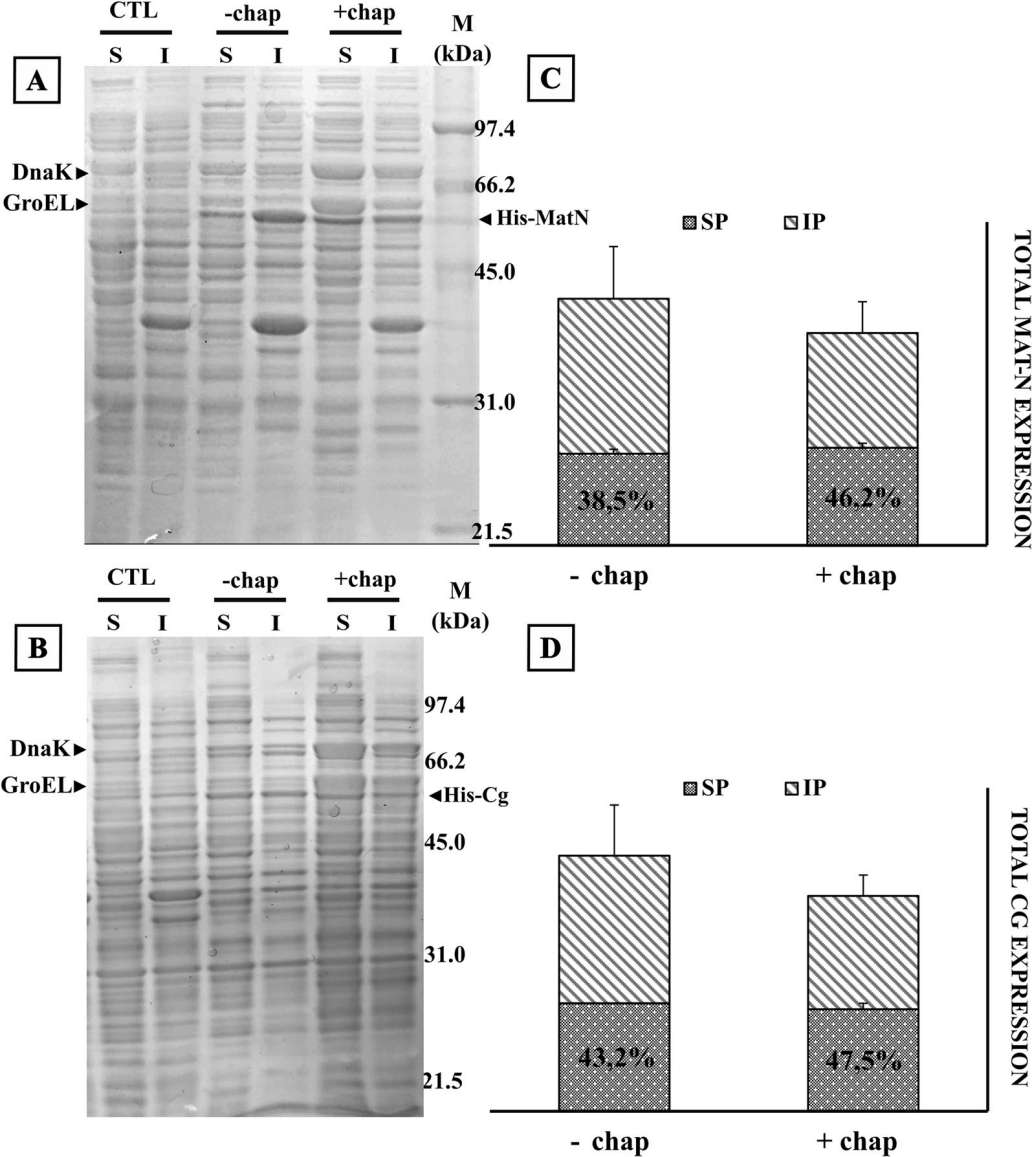
Co-expression of chaperones with MatN and Cg endolysins for solubility improvement. **A, B** *E. coli* C43(DE3) harboring pET-32a-MatN/Cg and chaperone (chap) encoding pG-KJE8 was induced at 20 °C by l-arabinose (0.25 mg/mL) and Tetracycline (5 ng/mL) 1 h before 1 mM IPTG addition. Cells were harvested after 20 h of incubation with constant shaking and processed to obtain soluble (S) and insoluble (I) fractions. Equivalent protein amounts (10 µg) of each sample were loaded on 12% SDS-PAGE. Upon gel electrophoresis, gels were stained with Coomassie Brilliant Blue R-250 and unstained following standard protocols. Control (CTL) lanes correspond to cellular lysates of uninduced *E. coli* C43(DE3) harboring pET32a-MatN/Cg. Black arrow heads indicate overexpression of endolysin, dnaK and groEL chaperones. **C, D** Total amounts of recombinant endolysin (column height) and the ratio of soluble/insoluble protein (SP, gray dotted pattern; IP, diagonal lines pattern) were calculated after background normalization with ImageJ, open-source image processing software. Endolysin expression and solubility were analyzed in biological triplicates. Error bars represent SD of mean values

Expression of proteins acting as chaperones during translation has been a field of research for several years, both at the level of understanding fundamental processes of native protein production in different biological systems as well as a way to increase the stability of recombinant proteins in various expression systems [23]. Chaperone proteins would help stabilize nascent polypeptides facilitating their correct folding into mature proteins. The availability of cloned sets of chaperones (DnaK and GroEL) in a plasmid compatible with our expression vector led us to test their co-expression along with our chosen endolysin clones. Our results showed that even when the mentioned chaperones were co-expressed with the endolysins MatN and Cg, a meager increase in the solubility of MatN and no noticeable increase in the solubility of Cg was seen (Fig. 6).

### Post-expression steps in endolysin production: impact of the use of detergents and osmolytes during the extraction process

Our results described above suggested that endolysin solubility could not be significantly influenced by modification of growth conditions, solubility tags and chaperones co-expression. Thus, we investigated the effects of adding detergents and different osmolytes during the cell pellet disruption [24, 25]. To this aim, we initially tested several detergents which are commonly used for bacterial protein extraction (Tween 20, Triton-X100, CHAPS, *N*-lauroylsarcosine, and Nonidet P40). We have also tested low molecular weight osmolytes such as polyalcohols, salts, and sugars (i.e., NaCl, CaCl_2_, mannitol, trehalose, glycerol, glycine betaine) which help to stabilize proteins. Our results showed a marginal improvement in the amount of extracted endolysin except for *N*-lauroylsarcosine when using detergents. Glycerol, trehalose or glycine betaine added to the lysis buffer increased the yield of soluble MatN endolysin two-fold while the effect on Cg endolysin was less pronounced (see Additional file 2).

**Fig. 7:**
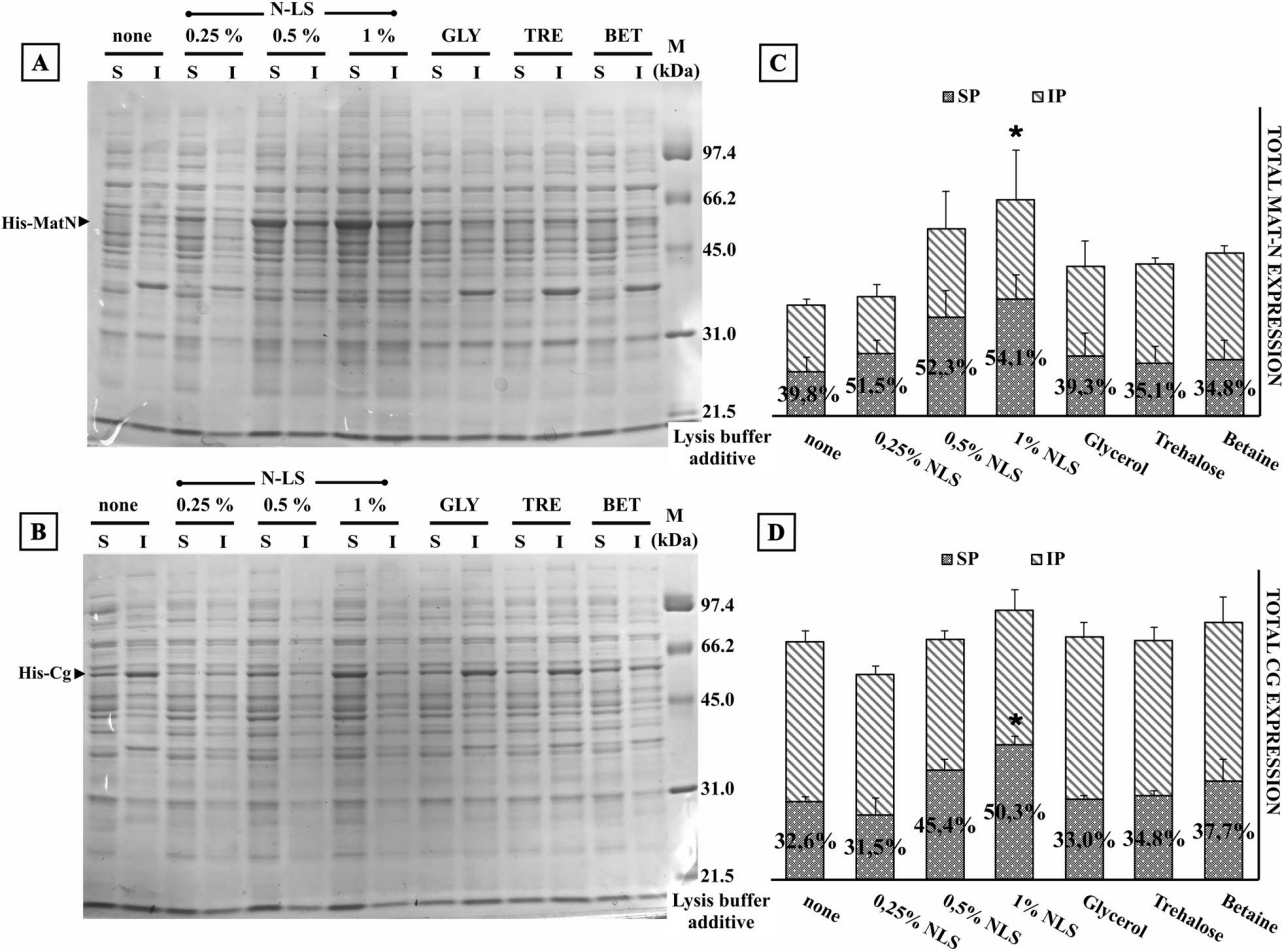
Impact of the use of detergents and osmolytes during the extraction process. **A, B** *E. coli* C43(DE3) harboring pET-32a MatN/Cg was induced at 20 °C by adding 1 mM IPTG. Cells were harvested after 20 h of incubation with constant shaking and processed to obtain soluble (S) and insoluble (I) fractions. Cell pellets lysis was performed by a bead-beating approach in lysis buffer (50 mM Tris–HCl pH 8.0, 150 mM NaCl, 1% glycerol, and 1 mM PMSF) supplemented with various concentrations of N-lauroylsarcosine (N-LS), 25% glycerol, 0.75 M trehalose or 1 M glycine betaine. Equivalent protein amounts (10 µg) of each sample were loaded on 12% SDS-PAGE. Upon gel electrophoresis, gels were stained with Coomasie Brilliant Blue R-250 and unstained following standard protocols. **C, D** Total amounts of recombinant endolysin (column height) and the ratio of soluble/insoluble protein (SP, gray dotted pattern; IP, diagonal lines pattern) were calculated after background normalization with ImageJ, open-source image processing software. Endolysin expression and solubility were analyzed in biological triplicates. Error bars represent SD of mean values. *p  <  0.05 (total MatN endolysin and ratio of soluble Cg endolysin vs. none additive group)

To confirm these observations, we performed new assays that included the best performing osmolytes and *N*-lauroylsarcosine at different concentrations (0.25; 0.5 and 1% w/v) (Fig. 7). Supplementation of the lysis buffer with *N*-lauroylsarcosine caused a strong increment in the soluble yield of the endolysins in a concentration-dependent manner. NLS at 1% w/v increased both total and soluble MatN recovery (*p* = 0.022, *p* = 0.047) and the soluble recovery of Cg (*p* = 0.005). The addition of 0.5% w/v *N*-lauroylsarcosine to the extraction buffer did not affect endolysin activity as judged by zymograms, thus supporting its use (Additional file 3). Osmolytes added at the protein extraction step only marginally improved the recovery of endolysins in the soluble fraction.

## Discussion

As part of a large sequencing project on *S. aureus* phage genomics, our group has isolated and sequenced more than 30 phages active on this pathogen, making available the sequence of their endolysins (Suárez et al., submitted to Microbiology Spectrum, Spectrum00334-22). Upon phylogenetic analysis, we expressed two of them, which were distant from an evolutionary standpoint. Yield of recombinant endolysins is of outmost importance when considering their industrial production. From that perspective, we adopted a systematic strategy addressing the expression (temperature and inducer concentration, cloning strategy, use of solubility tags and co-expression of molecular chaperones) or recovery (lysis buffer composition: osmolytes and detergents) steps.

Our results indicate that cloning strategy potentially impact in endolysin expression depending on the position of the insert (C-end or N-end) regarding the (His)_6_ tag, in agreement with previous reports [22].

As mentioned, production at the bench scale of soluble recombinant endolysins targeting *S. aureus* was reported by many groups [15, 17, 18, 26–33]; however, inspection of the published data did not reveal how much protein was lost in the insoluble fraction in each case and often, the extraction conditions and final yields were not stated (Table 1). A traditional and straightforward strategy to improve the solubility of recombinant proteins in bacterial expression systems consists of tuning down expression through low growth temperatures or reducing the concentration of the chemical inducer of the expression system. In this way, peptide crowding in the cytoplasm leading to insolubility could be prevented [34–36]. According to the literature as exemplified in Table 1, growth temperatures ranging between 10–25 °C were the most used ones, and yields—when stated—were very uneven ranging from 0.5 to 80 mg/L [18, 26, 30, 33]. In our hands, the highest yields, approximately 38–42% of soluble MatN and Cg (25 mg/L of induced culture), were obtained upon expression at 20 °C for 20 h using 1 mM IPTG.

Our attempts to increase solubility by the introduction of N-term fusion partners such as Trx (~ 12 kDa), SUMO (~11 kDa) and DsbC (~ 23 kDa) failed to produce the expected effect; in fact, we obtained the opposite result since total protein expression decreased when compared to the His-tagged constructs. These results are not only counter-intuitive but also have not been reported for any other recombinant protein [34, 37]. The strategy of adding solubility tags as means to increase the yield of endolysins is debatable, as most of those tags represent a 20–50% increase of the fusion protein molecular weight. Elimination of unwanted peptide sequences would also require the addition of an additional protease recognition site in the construct. Thus, taken as a whole, this approach may not be cost/benefit useful unless a large proportion of the synthesized endolysin is recovered in the soluble cell extract fraction.

In a different approach to improve soluble endolysin recovery, the co-expression of molecular chaperones (such as DnaK, GroEL and TF, reviewed in [23, 34]) was tested. These chaperones would prevent aggregation by acting on exposed hydrophobic patches of the recombinant endolysin, thus stabilizing misfolded or partially folded molecules. Disappointingly, this approach did not increase the amount of soluble endolysins to a convenient level. There are several reports pointing out that chaperones sometimes are by themselves responsible for undesirable side effects, from proteolysis to reduced yields or recombinant protein aggregation [38].

There are numerous environmental factors affecting the protein stability during cell lysis, such as type of buffer, pH, salt concentrations, presence of stabilizing agents (osmolytes, detergents), among others [24, 25]. These lysis media parameters or additives can either act by diminishing aggregation and protein–protein interactions or by stabilizing newly synthetized polypeptides. Interestingly, Filatova et al*.* [39] analyzed the effect of low molecular weight osmolytes and cations on the thermal stability of LysK; their results point out that dilution of the enzyme, addition of trehalose, sorbitol, glycerol, Mg^2+^ or Ca^2+^ increased LysK stability 100-fold. However, none of these factors have previously been evaluated as mediators of enhanced endolysin recovery. Thus, we tested variables that could be compatible with industrial scaling up. Our results showed that *N*-lauroylsarcosine was the most effective detergent at concentrations of 0.5–1.0 w/v, increasing the recovery of soluble MatN and Cg up to more than 50% of the total endolysin (Fig. 7). During the lysis of the cell pellet, both cytoplasmic and outer membrane components will interact with recombinant proteins, trapping them through hydrophobic and anionic surfaces. The detergent may prevent aggregation by disrupting associations with highly insoluble, aggregation-prone outer membrane components [40]. As described by Žydziecki et al. [16], there is a proven interaction between the positively charged endolysin and nucleic acids, in this aspect, the detergent may displace the nucleic acid avoiding endolysin intermolecular interactions.

In summary, our results taken as a whole suggest that the use of *N*-lauroylsarcosine during cell lysis, may increase the yields of soluble recombinant full-length endolysins.

## Conclusions

To our knowledge, this is the first systematic approach to evaluate factors influencing endolysins expression and extraction conditions. We found that low induction temperatures (20 °C) benefit the recovery of soluble enzyme and that over the range of 0.1–1 mM IPTG, there is no appreciable changes in endolysin yields. The position of the (His)_6_-tag in the polypeptide as well as extra amino acid residues produced by the cloning strategy may be potentially detrimental. Neither the use of fusions to solubility enhancers (Trx, DsbC and Sumo proteins) nor the co-expression of molecular chaperones increased the yield of recombinant endolysins. Our examination of the effect of adding tensioactives or osmolytes to the lysis buffer showed that 25% glycerol, 0.75 M trehalose and 1 M glycine betaine would benefit endolysins recovery but, anionic detergent *N*-lauroylsarcosine was the only addition making an visible difference in soluble protein yields with no impairment of enzymatic activity.

Clearly, there is plenty of room to improve expression of recombinant endolysins other than the ones approached in this work, namely alternative expression systems, host strains, different purification protocols, etc. However, we show here in that the use of lysis buffer additives (e.g., *N*-lauroylsarcosine), is a sensible strategy to increase the levels of soluble *S. aureus* endolysins.

## Methods

### Reagents, bacterial strains, media, and growth conditions

*E. coli* DH5α and *E. coli* C43(DE3) strains were used as cloning host and protein expression host, respectively. Strains were grown in Luria–Bertani (LB) medium at 37 °C with shaking at 150 rpm unless indicated otherwise. Ampicillin (Ap, 100 µg/mL) and Chloramphenicol (Cm, 20 µg/mL) were added when required.

### Endolysin cloning

The isolation, characterization and genomic sequencing of a set of bacteriophages active on *Staphylococcus aureus* will be reported elsewhere (Suárez et al., submitted to Microbiology Spectrum, Spectrum00334-22). Bioinformatics analysis using BLAST with LysK endolysin gene sequence as query allowed for the identification of 25 putative endolysin encoding genes. Cg and MatN endolysin encoding genes representing different phylogeny groups (41.53% identity, E = 5.10^–4^), as determined using MEGA 6.0 molecular evolutionary analysis software, were chosen for cloning and expression studies. DNA isolation of the chosen bacteriophages was carried out as previously described [41].

Cg and MatN encoding genes were cloned by Restriction Free Cloning (RFC) [42] into pET32a vector as described by Correa et al. [43]. This vector suite enables the parallel cloning of the same PCR product into different expression vectors harboring a selection of solubility fusion TAGs. The endolysin genes were fused as a C-terminal partner with (His)_6_-thioredoxin A (his_6_-Trx), (His)_6-_small ubiquitin-like modifier protein (his-SUMO), (His)_6-_disulfide bond isomerase C (his_6_-DsbC), or (His)_6_ tag alone. MatN gene was also cloned by restriction digestion and ligation (RD + L) into NdeI-BamHI digested pET-22b to generate a C-terminal (His)_6_ tag version of the recombinant endolysin. PET32a vector was also used when the impact of the expression of molecular chaperones on endolysin solubility was determined. All constructs were confirmed by sequencing. Primers used for endolysin cloning are listed in Table 2.

**Table 2:** primers used for endolysin cloning

Endolysin gene	Cloning method	PCR primers
MatN	RFC	Fw 5′-GGATCGGAAAACCTGTATTTTCAGGGATCCATGGAGGTGGCGACAATG-3′
		Rv 5′-GGTGGCTCCAGCTGCCGGATCCCTAACTGAT TTCTCCCCATAAGTCA-3′
Cg	RFC	Fw 5′-GGATCGGAAAACCTGTATTTTCAGGGATCCGTAATGGCTAAGACTCAAGCAG-3′
		Rv 5′-GTGGCTCCAGCTGCCGGATCCCTATTTGAATACTCCCCAGGCA-3′
MatN	RD + L	Fw 5′-GCGCATATGCAAGCAAAACTAACTA-3′
		Rv 5′-GGATCCGAGATTTCTCCCCATAAGTC-3′

### Endolysin expression

Flasks containing 10 mL of LB supplemented with the appropriate antibiotics were inoculated with an aliquot (1/100 v/v) of stationary cultures of *E. coli* C43(DE3) harboring pET-32a-Cg or pET32a-MatN plasmids. Cultures were incubated at 37 °C with constant shaking (150 rpm) until they reached OD_600_ = 0.6–0.8. Induction was started by the addition of various IPTG concentrations (ranging from 0.1 to 1 mM), and growth was continued for 20 h at 20 °C, 25 °C or 30 °C. Induced cells (10 mL) were harvested by centrifugation at 3500*g* for 5 min at 4 °C and the pellet was re-suspended in 1 mL of lysis buffer (50 mM Tris–HCl pH 8.0, 150 mM NaCl, 1% glycerol and 1 mM PMSF). Disruption of cells was accomplished using a bead beater homogenizer (Fast-Prep^®^) following 6 cycles of 15 s bursts and 2 min rest on ice protocol. Lysates were centrifuged at 16,000*g* for 30 min at 4 °C to separate soluble and insoluble fractions; insoluble pellets were re-suspended thoroughly in 250 µL lysis buffer and both fractions were either processed immediately or kept frozen at − 80 °C until use.

### Cellular lysate analysis

Cellular lysates were analyzed by 12% sodium dodecyl sulfate–polyacrylamide gel electrophoresis (SDS-PAGE). Protein concentration was determined by Bradford Coomassie Brilliant Blue assay [44] to ensure loading of equivalent protein mass (10 µg) on each lane. Upon gel electrophoresis, gels were stained with Coomasie Brilliant Blue and unstained following standard protocols [45]. Following image acquisition, changes in soluble endolysin content under each experimental condition were visualized and quantitated using ImageJ (open-source image processing software). The ratio of soluble/total recombinant endolysin was calculated after normalization with background values obtained from the pixel densities of bands corresponding to endogenous proteins. Unless indicated otherwise, endolysin expression and solubility was analyzed in biological triplicates.

### Chaperone co-induction assay

The plasmid pG-KJE8 (TAKARA BIO INC) encoding chaperones (dnaK and groEL) and their co-factors (dnaJ-grpE-groES), was transformed into the expression strain *E. coli* C43 (DE3) harboring the chosen endolysin plasmid. Co-expression of chaperones was induced by L-arabinose (0.25 mg/mL) and Tetracycline (5 ng/mL) 1 h before IPTG addition. Once induced, cultures were grown with shaking for 20 h at 20 °C, harvested by centrifugation at 3500*g* for 5 min at 4 °C and the pellet was re-suspended in 1 mL of lysis buffer.

### Solubility tags expression assay

Endolysin genes were cloned using the RFC approach in a series of modified pET-32a vectors [43] that enables cloning of fragments as fusion with a selection of solubility tags with a single megaprimer pair. Oligonucleotides for megaprimer generation were designed according to Bond and Naus [42]. In brief, the amplification conditions were as follows: 30 s at 98 °C followed by 30 cycles of 98 °C for 10 s, 62 °C for 30 s and 72 °C for 30 s with a final extension step at 72 °C for 5 min. Megaprimers were purified by extraction from agarose gel. The integration of megaprimers into the vectors were done under the following reaction parameters: 30 s at 98 °C and 25 cycles of 98 °C for 10 s, 60 °C for 30 s and 72 °C for 5 min with a final extension step at 72 °C for 7 min. Subsequently, an aliquot (10 µL) of the reaction mixture was digested with 1 µL of DpnI (Thermo Scientific) ON at 37 °C in order to remove the parental plasmid, and this was transformed into 100 µL of competent DH5α *E. coli* cells.

### Lysis buffer supplementation for solubility enhancement

Buffer supplementation with osmolytes or detergents were tested as solubility enhancers by incorporation of each one at the following concentrations into the standard lysis buffer before cell disruption**:** 300 mM NaCl, 25% v/v glycerol, 0.5 M mannitol, 0.75 M trehalose, and 1 M glycine betaine; detergents were added at 0.5% v/v in the case of Tween 20, Triton-X100 or Nonidet P40; or 0.5% w/v in the case of CHAPS or *N*-lauroylsarcosine.

### Statistical analysis

Statistical analysis was performed using JASP (Version 0.11, JASP Team, 2019). Normal distribution of data sets was evaluated through Shapiro–Wilk test, designed to test normality for small data-size, and homoscedasticity was judged by Levene's test. The two tailed Student t-test was used when comparing means from two experimental groups, whereas analysis of variance (ANOVA) followed by Tukey’s *post-hoc* test was chosen for multiple comparisons. When criteria of Normal distribution or equality of variances were not met, we applied non-parametric tests. Statistically significant differences are denoted by asterisks (*p < 0.05, **p < 0.005).

## Supplementary Information

**Additional file 1**: Hydrophobicity plots by the Kyle-Doolittle scale. Endolysin hydrophobic regions prediction.

**Additional file 2**: Impact of different detergents and osmolytes on soluble Cg and MatN endolysin recovery. Lysis buffer additives screening

**Additional file 3**: Zymograms of purified endolysins. SDS–Polyacrylamide gels containing autoclaved RN 4220 *S. aureus* cells were used to visualize the peptidoglycan hydrolyzing activity of recombinant His-MatN and His-Cg extracted in lysis buffer with 0.5% w/v N-Lauroylsarcosine or without the detergent.

## Data Availability

The datasets used and/or analyzed during the current study are available from the corresponding author on reasonable request.

## Abbreviations

RFC: Restriction free cloning
PG: Peptidoglycan
CBD: Cell binding domain
EAD: Enzymatically active domain
PMSF: Phenylmethylsulfonyl fluoride
PBS: Phosphate buffer saline
DTT: Dithiothreitol
