# Supplementary material for: Evaluation of factors influencing expression and extraction of recombinant bacteriophage endolysins in Escherichia coli
Source: Microb Cell Fact. 2022 Mar 15;21:40. doi: 10.1186/s12934-022-01766-9 (PMC8922839; doi:10.1186/s12934-022-01766-9)
Supplement: Supplementary file 1 — Additional file 1: Hydrophobicity plots by the Kyle-Doolittle scale. Endolysin hydrophobic regions prediction. [file 12934_2022_1766_MOESM1_ESM.pdf]

# ADDITIONAL FILE 1

**A-**MatN (CHAP- ami2-SH3)

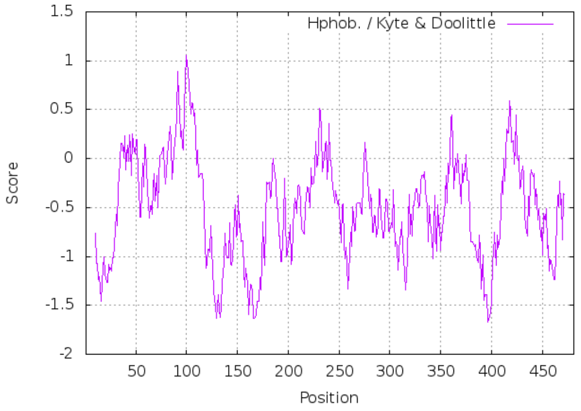


**B-**Cg (CHAP-ami2-SH3)

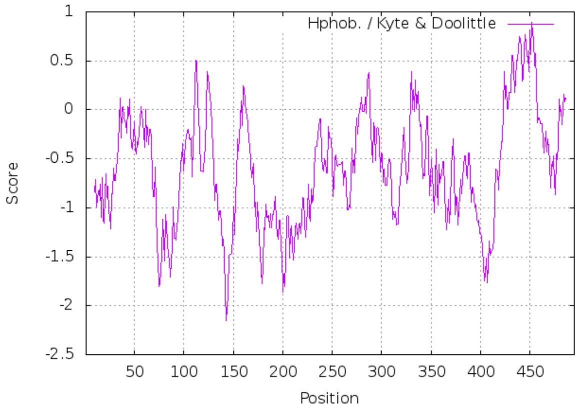


**C-**308 (CHAP – CBD non-SH3)

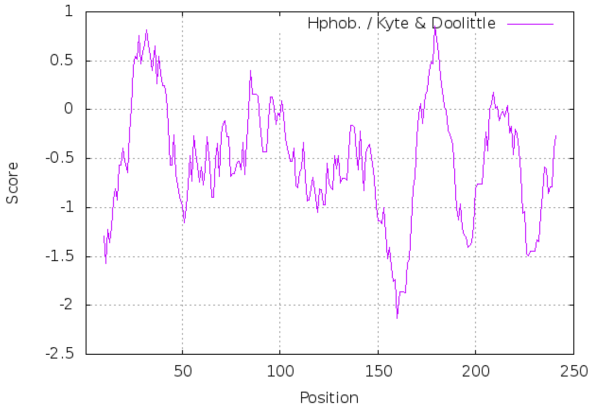


**D-**320 (CHAP- ami3-SH3)

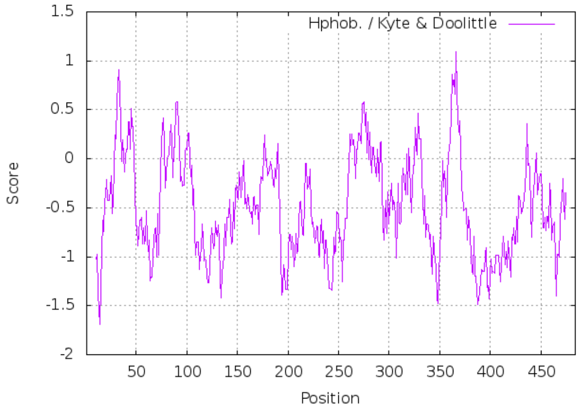


**E-**GH15 (CHAP-ami2-SH3)

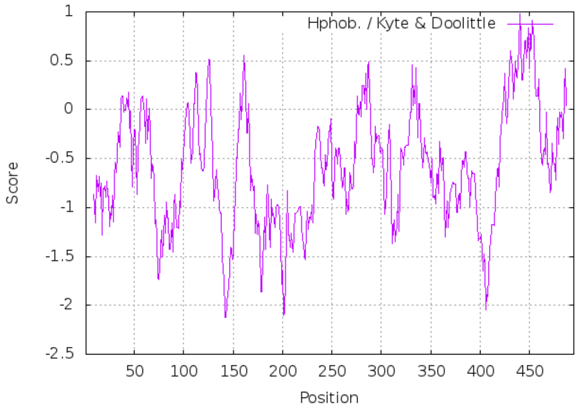


**F-**PlyGRCS: CHAP-SH3

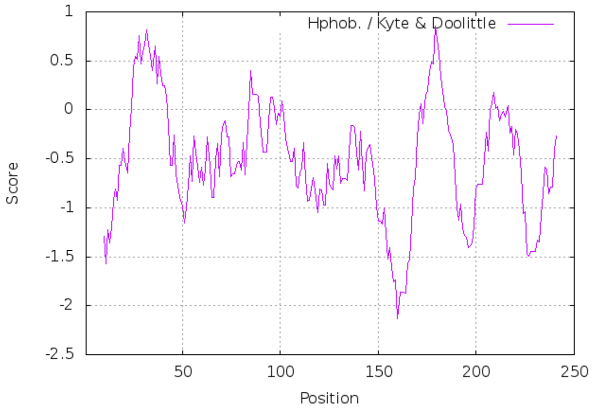


**G-**LysK (CHAP-ami2-SH3)

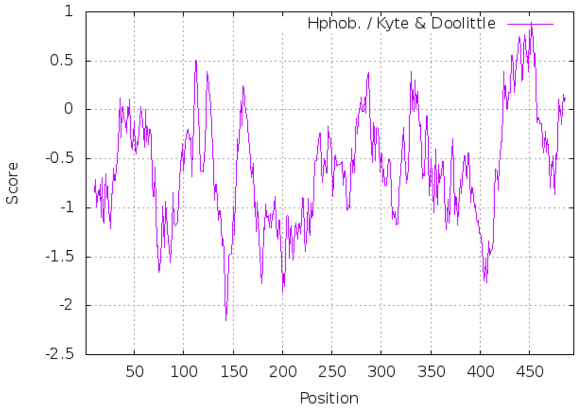


**H-**LysH5 (CHAP-ami2-SH3)

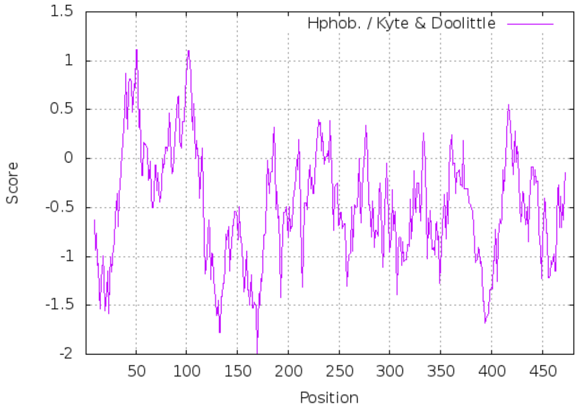

**Hydrophobicity plots of endolysins.** Kyte-Doolittle plot predicts whether different protein segments would interact with or reside in a membrane. Hydrophobic regions achieve positive values of at least 1.6 at their centers. The online tool ProtScale (https://web.expasy.org/protscale/) was used for the construction of plots. Protein sequences (A-D) were from phage endolysins sequenced in our laboratory (Suárez *et al*, submitted to Microbiology Spectrum, Spectrum00334-22) or reported in the literature (E-H) [17,29,14,18]. Endolysin domain composition is shown between brackets.
