# Supplementary material for: Evaluation of factors influencing expression and extraction of recombinant bacteriophage endolysins in Escherichia coli
Source: Microb Cell Fact. 2022 Mar 15;21:40. doi: 10.1186/s12934-022-01766-9 (PMC8922839; doi:10.1186/s12934-022-01766-9)
Supplement: Supplementary file 2 — Additional file 2: Impact of different detergents and osmolytes on soluble Cg and MatN endolysin recovery. Lysis buffer additives screening [file 12934_2022_1766_MOESM2_ESM.pdf]

ADDITIONAL FILE 2

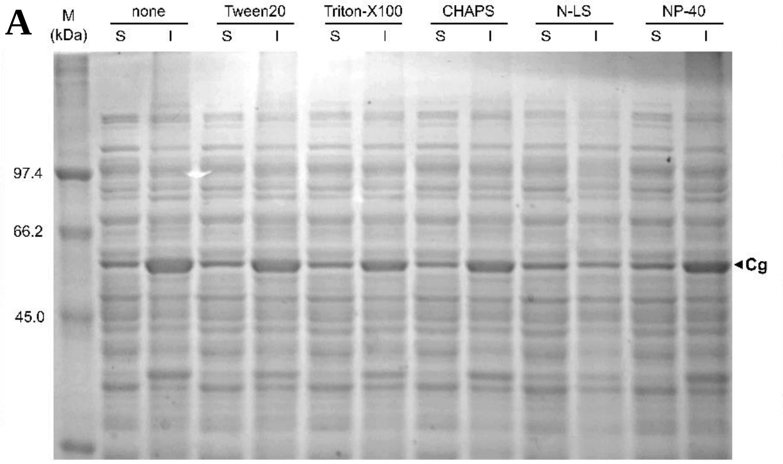

Detergent	S+I	% soluble
none	3,64	27,48
Tween-20	3,14	31,82
Triton X-100	2,57	36,20
CHAPS	2,71	28,83
NLS	1,82	**57,18**
NP-40	3,41	30,86

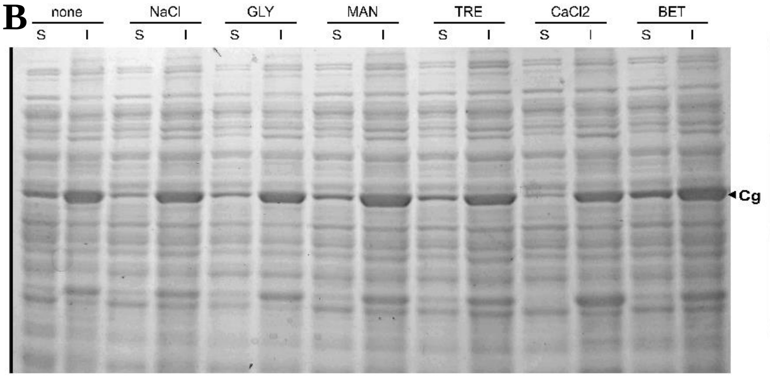

Osmolyte	S+I	% soluble
none	3,64	27,46
NaCl	2,80	25,88
Glycerol	3,48	28,76
Mannitol	2,70	28,99
Trehalose	2,44	34,90
$CaCl_2$	1,76	17,37
Betaine	3,22	38,03

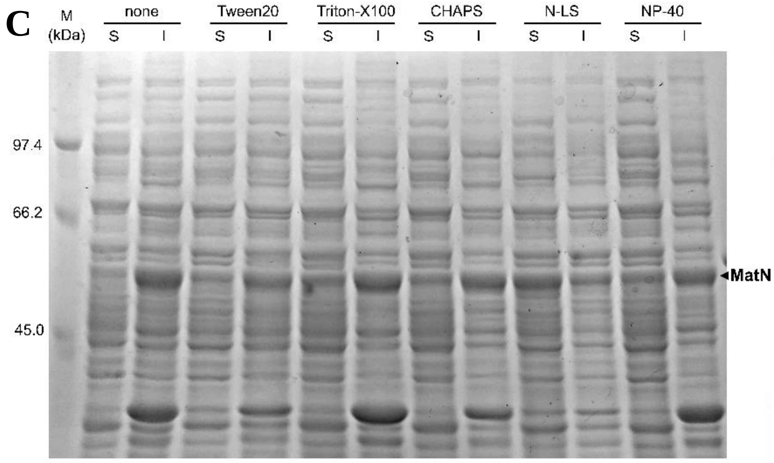

Detergent	S+I	% soluble
none	4,59	21,81
Tween-20	3,77	32,62
Triton X-100	4,91	29,96
CHAPS	4,53	34,88
NLS	4,97	**68,81**
NP-40	3,73	26,23

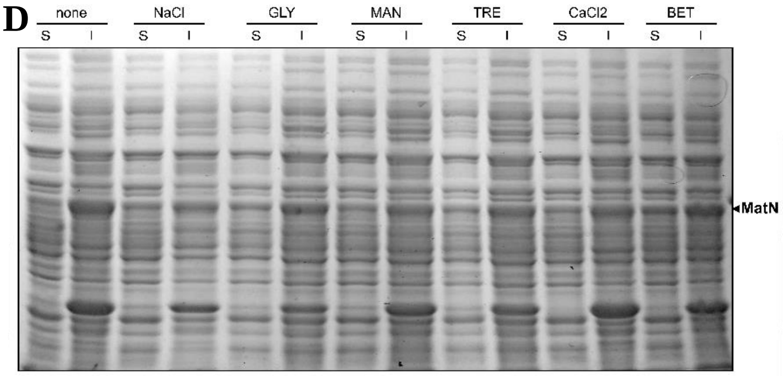

Osmolyte	S+I	% soluble
none	4,63	21,61
NaCl	4,65	27,95
Glycerol	4,63	**46,45**
Mannitol	3,74	36,96
Trehalose	3,42	**49,34**
$CaCl_2$	4,64	27,28
Betaine	4,31	**45,33**

Impact of different detergents and osmolytes on soluble Cg and MatN endolysin recovery. Detergents (0.5% v/v Tween 20, Triton-X100 or Nonidet P40; 0.5% w/v CHAPS or N-lauroylsarcosine) [panels A and

C] and osmolytes (300 mM NaCl, 25% glycerol, 0.5 M mannitol, 0.75 M trehalose, 5 mM $CaCl_2$, 1 M glycine betaine) [panels B and D] were added to the lysis buffer prior to cell pellet disruption by bead beater homogenizer. Upon SDS-PAGE and image acquisition, the ratio of soluble (S)/total recombinant endolysin (S+I) was calculated.
