# Supplementary material for: Evaluation of factors influencing expression and extraction of recombinant bacteriophage endolysins in Escherichia coli
Source: Microb Cell Fact. 2022 Mar 15;21:40. doi: 10.1186/s12934-022-01766-9 (PMC8922839; doi:10.1186/s12934-022-01766-9)
Supplement: Supplementary file 3 — Additional file 3: Zymograms of purified endolysins. SDS–Polyacrylamide gels containing autoclaved RN 4220 S. aureus cells were used to visualize the peptidoglycan hydrolyzing activity of recombinant His-MatN and His-Cg extracted in lysis buffer with 0.5% w/v N-Lauroylsarcosine or without the detergent. [file 12934_2022_1766_MOESM3_ESM.pdf]

ADDITIONAL FILE 3

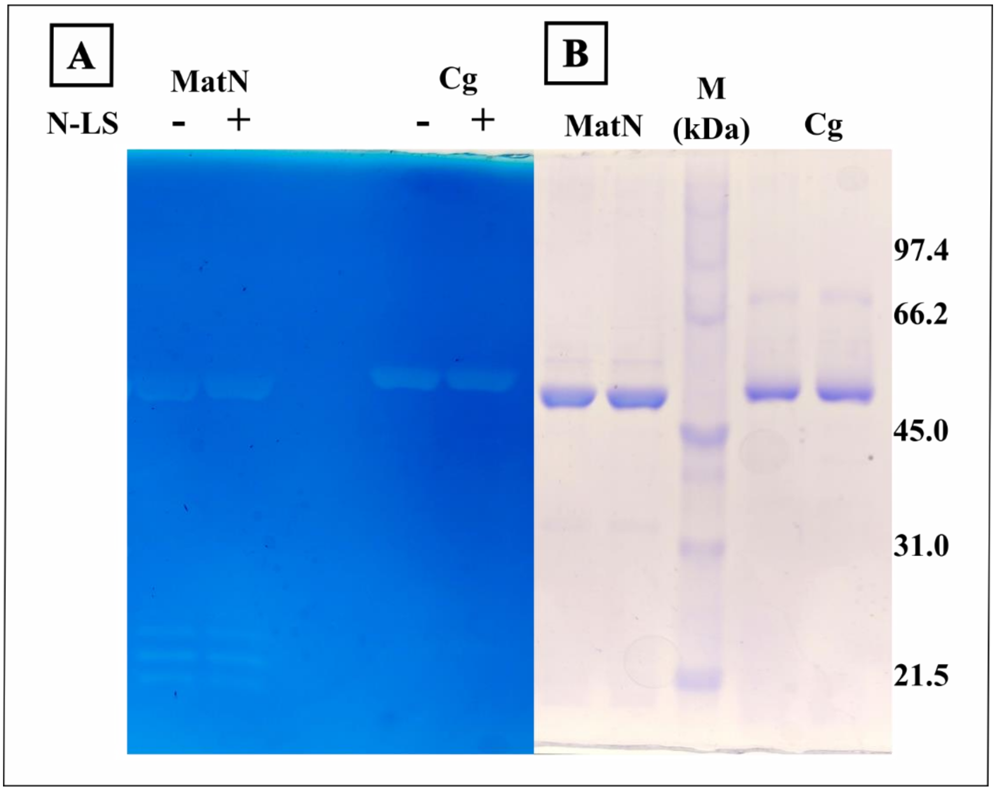

Zymograms of purified endolysins. SDS-Polyacrylamide gels containing autoclaved RN 4220 *S. aureus* cells were used to visualize the peptidoglycan hydrolyzing activity of recombinant His-MatN and His-Cg extracted in lysis buffer in the absence (-) or presence (+) of 0.5% w/v N-Lauroylsarcosine (N-LS).
